# Toward Providing Equitable Care for Adults with Congenital Heart Disease: Where We Are and Where We Need to Be

**DOI:** 10.1007/s11886-025-02307-7

**Published:** 2025-12-03

**Authors:** Jeannette P. Lin, Ariane Marelli, Aihua Liu, Liming Guo, Jennifer Desalvo, Danielle Hile, Mark Roeder, Jamil Aboulhosn, Curt J. Daniels

**Affiliations:** 1 University of California Los Angeles, Los Angeles, CA USA; 2 Institute of Circulatory and Respiratory Health, Canadian Institutes of Health Research McGill University, Montréal, QC USA; 3 Montreal Centre for Outcomes Research and Evaluation, The Research Institute of McGill University Health Centre, Montréal, QC Canada; 4 The Ohio State University Medical Center Nationwide Children’s Hospital , 452 W 10th Ave, Columbus, OH 43210 Canada; 5 Adult Congenital Heart Association, Media, PA USA

**Keywords:** Adult congenital heart disease, Work force, Epidemiology, Cardiology training, Care models

## Abstract

**Purpose of the Review:**

To understand the growth of the adults with congenital heart disease (ACHD) population and ACHD work force needs in the United States, and to consider solutions to provide access for this population.

**Recent Findings:**

The ACHD population is growing rapidly and is outpacing current efforts to improve access and delivery of ACHD care.

**Summary:**

Achieving adequate access for the ACHD population will require a multipronged approach, including training more ACHD cardiologists and advanced practice providers, expanding use of telehealth, increasing collaboration between larger tertiary ACHD care centers and smaller ACHD clinics and cardiology practices, and reimagining care models and pay structures.

## Introduction

Survival to adulthood for congenital heart disease (CHD) patients has increased due to improvement in pediatric cardiology care and congenital cardiac surgical techniques. A 2014 epidemiologic study utilizing population-based data in Quebec reported that in 2010, adults with congenital heart disease significantly outnumbered pediatric cases, accounting for roughly two-thirds of the total CHD population [1]. The prevalence of adults with CHD (ACHD) will continue to increase for the next several decades [2, 3]. Upon reaching adulthood, ACHD patients have higher risk for morbidity and mortality than those without CHD [4–8]. Specialized ACHD care improves clinical outcomes and improves survival [9, 10]; however, only a small percentage of ACHD patients have access to specialized care due to the inadequate numbers and current geographic distribution of ACHD cardiologists and ACHD care centers. Over the last several decades, efforts have been made to increase and improve the training of ACHD cardiologists, but the care gap remains. As the ACHD population continues to grow, will the growth and trajectory of this population outpace the efforts to expand the ACHD workforce and widen the care gap even further? If so, what strategies should be employed to address current and future ACHD patient care needs? 

In this review, we will explore:

1. The current and projected ACHD population.
2. The current and projected ACHD workforce, including ACHD cardiologists and ACHD care centers.
3. Current state of ACHD care delivery in the US.
4. Define the gaps in care in terms of workforce challenges, care delivery models, and geographical barriers to access.
5. Proposed interventions to close the care gap and provide adequate access to ACHD care across the US.

## Epidemiology of ACHD

According to an analysis of the Quebec CHD database, the prevalence of CHD in adults in Quebec exceeded that of children in 2000 [1]. As the prevalence of children with CHD remains static but survival of adults with CHD improves, ACHD patients will comprise a larger proportion of the overall CHD population in the coming decades. Notably, those with more severe forms of CHD survive to adulthood at a greater rate than in the past [2, 3]. For example, using data collected from 11 countries including the US, Australia and several European countries, Plappert and colleagues demonstrated an expected 20% rise in the prevalence of the patients with Fontan palliation over the next decade [11].

In the US, there are no data to provide an accurate estimate of the prevalence of ACHD patients. The Centers for Disease Control (CDC) was authorized by the US government though the Congenital Hearts Future Act to provide data on the US CHD population. But the US healthcare system lacks a centralized database, and hospital records may not be accurate or complete; thus, studies to date have not produced clear results [12]. As Canada has a universal health system and each patient is assigned a unique identifier that can be used to track diagnosis and utilization of health care services, Canadian administrative data on incidence of CHD and survival of CHD patients has been used to estimate the US ACHD population. In 2016, Gilboa et al. used published a study using Canadian data estimating a prevalence of 6.16 adults with congenital heart disease per 1,000 individuals, thus 1.4 million adults with CHD in the US in the year 2010 [13].

**Fig. 1:**
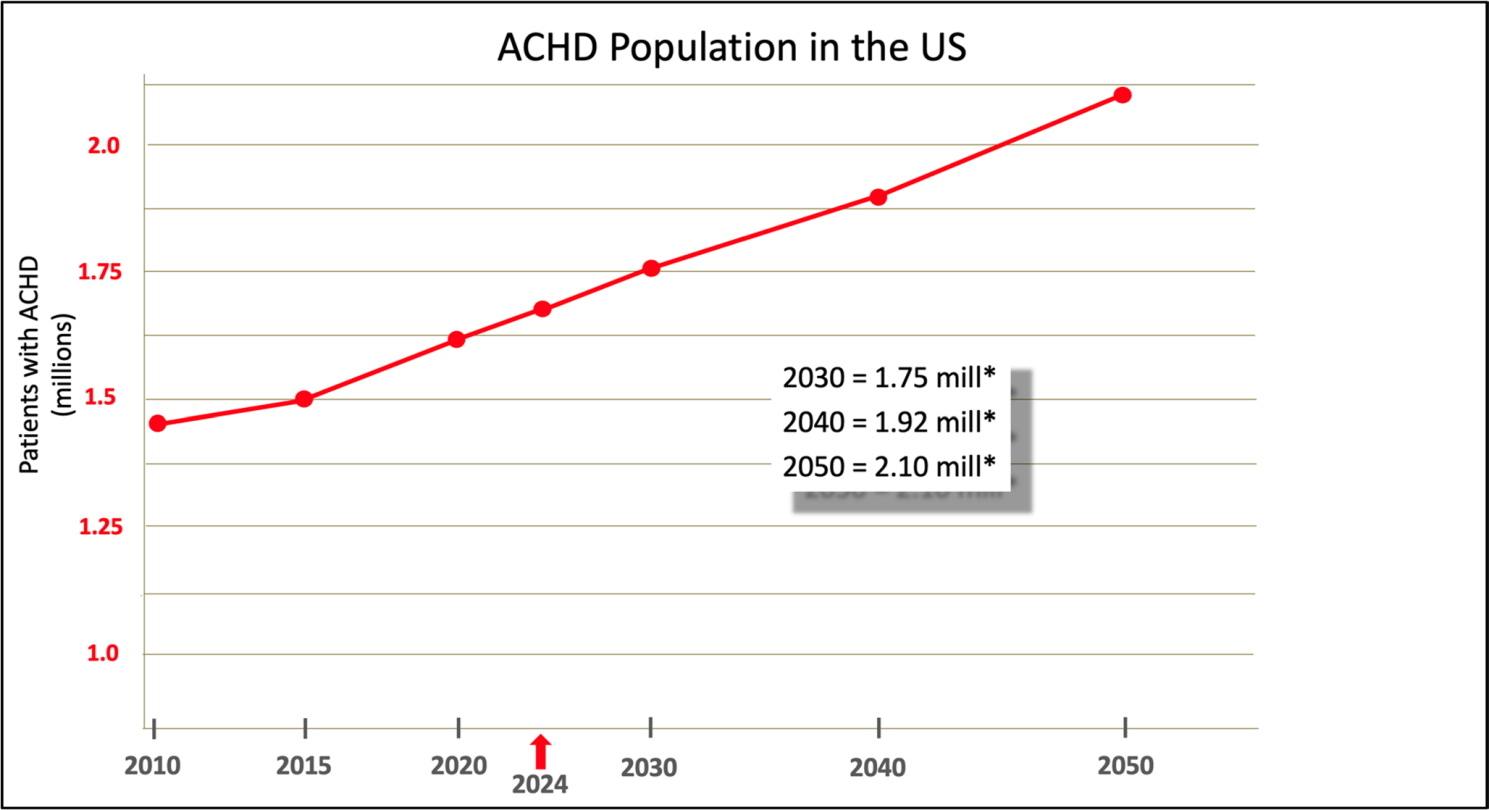
Estimated United States population of adults with congenital heart disease between 2010 and 2050 [13]

Using a conservative prevalence estimate of 6.0 adults with CHD per 1,000 population in 2020 and projecting an increase of 0.4% per year using the Quebec CHD database, the prevalence of adults with CHD is estimated to be 6.24 per 1000 in 2030, 6.50 per 1000 in 2040, and 6.75 per 1000 in 2050. We therefore estimate a point prevalence of 1.69 million adults with CHD in the US in 2024. Based on population growth, the projected point prevalence is 1.75 million in 2030, 1.92 million in 2040, and 2.10 million in 2050 (Fig. 1). 

## ACHD Work Force: ACHD Training and Board Certification

### History and Current State of ACHD Training

In 2001, the 32nd Bethesda ACHD Care Conference defined the need for training and education in ACHD [14]. Over the next decade, cardiologists in the US trained in ACHD through one of several pathways. The only pathway formally approved by the Accreditation Council for Graduate Medical Education (ACGME) was a 5 or 6-year program that provided dual training in both adult and pediatric cardiology. There were also several non-ACGME accredited pathways: (1) 1 or 2 years of dedicated ACHD training after adult cardiology or pediatric cardiology fellowship, (2) 1 year of ACHD training after adult or pediatric cardiology fellowship combined with advanced training in another discipline, i.e., ACHD/echocardiography, or (3) dedicated months of ACHD experiences during adult or pediatric cardiology fellowship without additional time after adult or pediatric cardiology fellowship. Between 1987 and 2011, 63 cardiologists completed one of these ACHD training pathways.

In 2012, ACHD cardiologists proposed formalizing ACHD fellowship training with monitoring and accreditation by the ACGME, and developing a board certification in ACHD through the American Board of Internal Medicine (ABIM) in cooperation with the American Board of Pediatrics (ABP). The ACGME Program Requirements for ACHD were developed by an expert panel of experienced pediatric and ACHD cardiologists. The ACGME-accredited ACHD fellowship requires a 24-month training program: 18 months of clinical training and 6 months of electives and/or research. Completion of an either pediatric cardiology or adult cardiology fellowship is required prior to starting ACHD fellowship. In 2015, the first ACHD fellows began training in fellowships accredited by the ACGME.

Between 2015 and 2019, the 10–15 existing ACHD training programs were required to bring their training programs into compliance with the requirements outlined in the ACGME Program Requirements for Adult Congenital Heart Disease [15] and apply for ACGME accreditation. In the years that followed, new ACHD fellowship programs were also developed at centers that did not previously offer ACHD training. The number of ACGME-accredited ACHD fellowship programs increased from 11 programs in 2017 to 32 programs in 2025. The number of available fellowship positions in the National Residency Matching Program (NMRP) increased from 9 positions in 2018 to 30 positions in 2024, reflecting a significant increase in training opportunities. The number of applications for ACHD fellowships received in the Electronic Residency Application System (ERAS) averaged 17.7 between 2019 and 2023; in 2024, applications increased to 32. Over the past 5 years (2020–2024), there have been an average of 14.8 ACHD fellowship graduates per year.

Although the number of ACHD fellowship programs and positions continues to exceed the number of ACHD applicants each year, the growth of ACHD fellowship programs and positions has been critical to the growth in number of ACHD graduates for two important reasons. First, the presence of an ACHD fellowship program - and thus ACHD fellows - at an institution increases the visibility of the specialty within the institution, providing ACHD exposure for medical students and residents that might not otherwise be aware of ACHD as a unique discipline. Second, ACHD fellowship represents the 7th and 8th year of postgraduate training for those who completed pediatrics or internal medicine residencies, and the 8th and 9th year of postgraduate training for those who completed combined internal medicine and pediatrics (Med-Peds) residencies. Many trainees have personal responsibilities that prevent them from being able to relocate to pursue additional training. Having geographic diversity in ACHD fellowships increases the opportunities for individuals to pursue ACHD training while decreasing the need for trainees to relocate to a different geographic region.

### History and Current State of ACHD Board Certification

In 2015, the first ACHD board exam was administrated by the ABIM. For the first three ACHD board exams in 2015, 2017, and 2019, cardiologists could attain eligibility for the board exam by completing a 2-year ACGME-accredited fellowship (“Training Pathway”), or by certifying that they were actively practicing ACHD for a minimum required percentage of their clinical time (“Practice Pathway”). From 2021 onwards, only those who have completed an ACGME-accredited ACHD fellowship program are eligible to take the ABIM ACHD board examination.

**Fig. 2:**
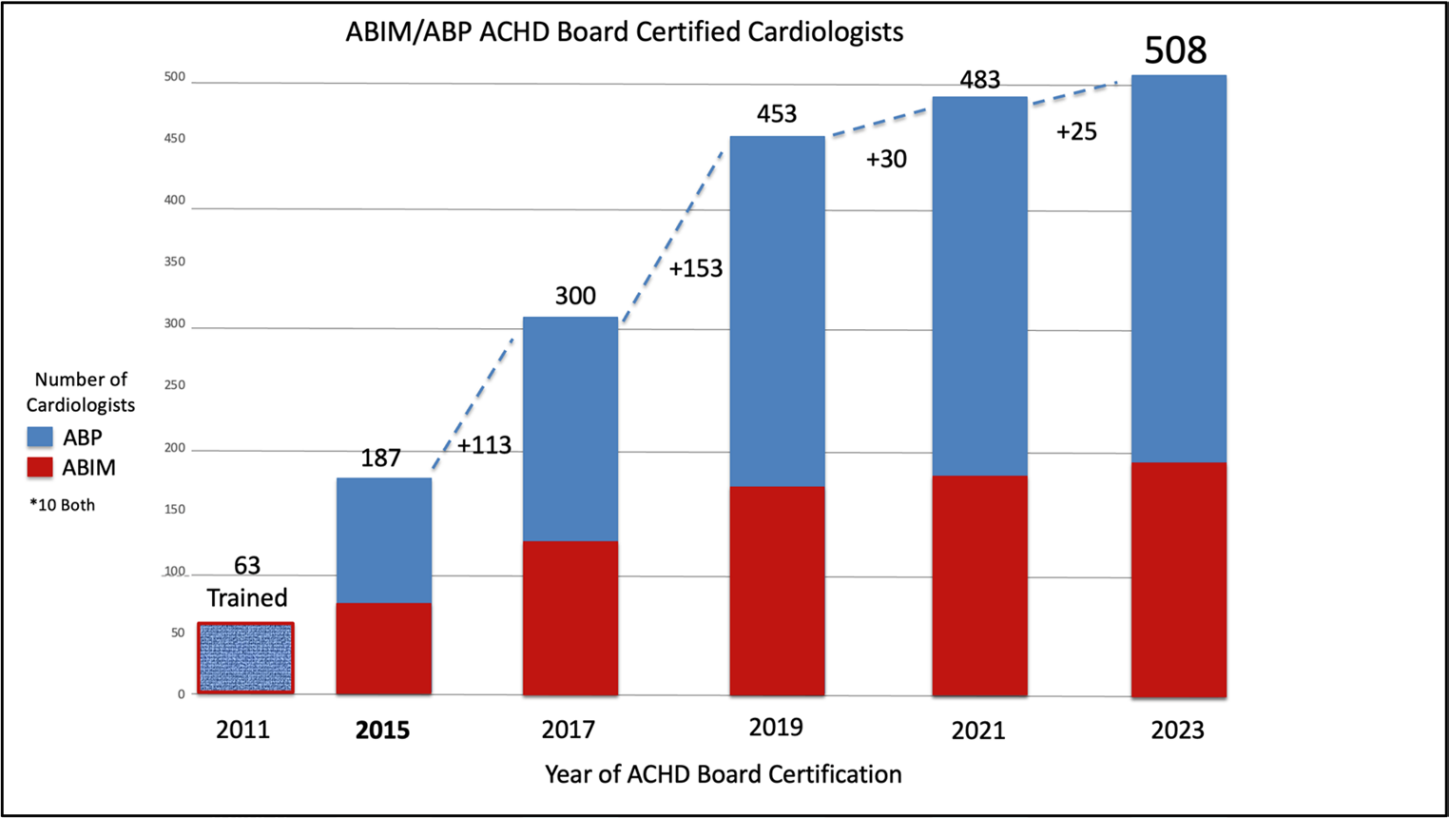
ABIM/ABP ACHD Board Certified Cardiologists by exam year. Prior to the ACGME fellowship and the first ABIM ACHD in 2015, 63 cardiologists had completed some level of training. ABP represents those with a pediatric cardiology background and ABIM those with IM cardiovascular background. A total of 508 have completed ACHD board certification with last exam in 2023. ABP = American Board of Pediatrics, ABIM = American Board of Internal Medicine; ACHD = adults with congenital heart disease

In 2024, there were 508 ACHD board certified cardiologists in the US (Fig. 2). As cardiologists can become eligible for ACHD board certification only by completing an ACGME-accredited ACHD fellowship, the size of the work force is thus dependent on number of fellowship applicants, number of fellowship positions, and fellowship graduation rates.

## Current State of ACHD Care Delivery in the United State: ACHD Clinics and Centers

Care delivery models for ACHD have evolved over the last few decades. The 32nd Bethesda ACHD Care Conference in 2001 published the first consensus recommendations for ACHD programs based on the multidisciplinary needs of ACHD patients [14].

### ACHD Clinics and Centers

To assist ACHD patients seeking ACHD care, the Adult Congenital Heart Association (ACHA) began publishing an ACHD Clinic Directory on its website in 2010. ACHD clinics could be included in the directory by submitting their clinic information to the ACHA.

**Fig. 3:**
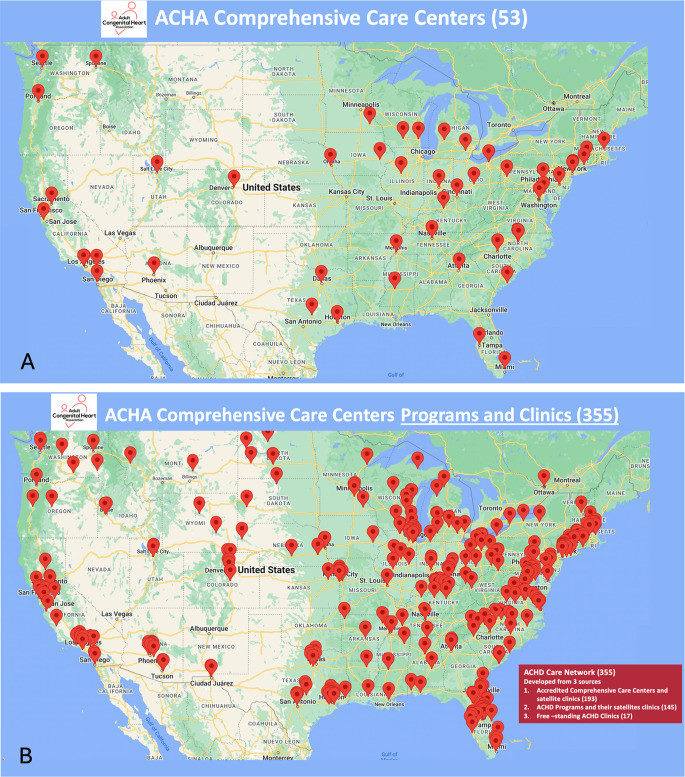
Adult Congenital Heart Association (ACHA) Comprehensive Care Centers (**A**); ACHA Comprehensive Care Centers and Clinics (**B**) [17]

In 2012, the ACHA convened an expert panel to develop criteria for ACHA accreditation of ACHD programs. After vetting with ACHD patients, families and professional advocacy organizations, the criteria were finalized in 2015. Approval as an accredited center requires meeting detailed program requirements [16] and successfully completing a site visit. The criteria recognize that delivery of specialty care requires appropriate resources as well as cardiologists with expertise in the care of ACHD patients, partnerships with surgeons, electrophysiologists, heart failure experts, advanced practice providers (APP), obstetricians/gynecologists, radiologists, and social workers. In 2017, there were 17 sites approved as ACHA-accredited ACHD Comprehensive Care Centers (ACHD CCC), expanding to 53 ACHA-accredited ACHD CCC in 2024 (Fig. 3A). ACHA-accredited ACHD CCC are designated as such in the ACHA Clinic Directory [17]. The remainder of programs in the ACHA ACHD Clinic Directory are designated as “ACHD Clinics.” In 2024, there were 302 ACHD Clinics listed in the ACHA ACHD Clinic Directory. Together with the 53 ACHA-accredited ACHD CCC, there are a total of 355 ACHD CCC and ACHD Clinics in the US providing access to ACHD care (Fig. 3B).

Despite more limited scope of services, ACHD Clinics provide an important regional access point for patients. ACHD Clinics are typically able to perform clinical evaluation and diagnostic tests for ACHD patients, but may not have the resources, staffing and capability to perform complex ACHD surgery and/or catheter-based interventions. ACHD Clinics will typically refer to an ACHD CCC for services they are not able to provide within their own program.

An important limitation of the current review is that there are no national data demonstrating where ACHD patients are receiving care, and ACHA accreditation as an ACHD CCC or listing as an ACHD Clinic is not required for provision of care to ACHD patients or reimbursement for ACHD care by any insurance programs. Thus, although ACHA ACHD Clinic Directory is being used in the current analysis to identify ACHD centers, there may be clinics providing ACHD care that are not listed in the directory. This may result in undercounting ACHD clinics and overestimation of the ACHD care gap. Conversely, there may be closures of listed ACHD Clinics that are not updated in the ACHA ACHD Clinic Directory, resulting in overcounting of ACHD clinics and underestimation of the ACHD care gap. Nonetheless, the ACHA ACHD Clinic Directory remains the most reliable source of data about ACHD programs; for the purposes of this analysis, we will regard programs as either ACHA-accredited ACHD CCC or ACHD Clinics based on their listing in the ACHA ACHD Clinic Directory.

## Determining the Care Gap for Patients with ACHD

To determine the “Care Gap” – the gap between the needs of ACHD patients and the ability of the ACHD workforce to care for ACHD patients in the US - we need to consider a number of factors, including: (1) the number of ACHD patients outlined above, (2) the number of ACHD providers, (3) the number of ACHD patients seen by each ACHD provider, (4) ACHD care delivery models, and (5) the geographic distribution of patients and providers. In this study, we present the following indices to quantify the care gap: the patient to provider ratio, the number of patients who need care but have no access to care, the number of providers/care centers needed to provide adequate care.

There are no data on how many patients an ACHD cardiologist can care for, as this depends on several variables, including (1) presence and number of dedicated administrative and clinical staff, (2) practice setting in a large academic or non-academic center, (3) percentage of provider time spent in clinical practice caring for ACHD patients, and (4) complexity of the patient population. Data from the ACHA accreditation program estimates a ratio of 830 patients per ACHD board certified cardiologist at an ACHD CCC. Marelli and colleagues estimated an ACHD cardiologist within an ACHD Center would care for 950 patients [18]. Therefore, it would seem reasonable that ≤ 1000 patients per ACHD board certified cardiologist is a target benchmark for an ACHD cardiologist practicing at an ACHD CCC.

**Fig. 4:**
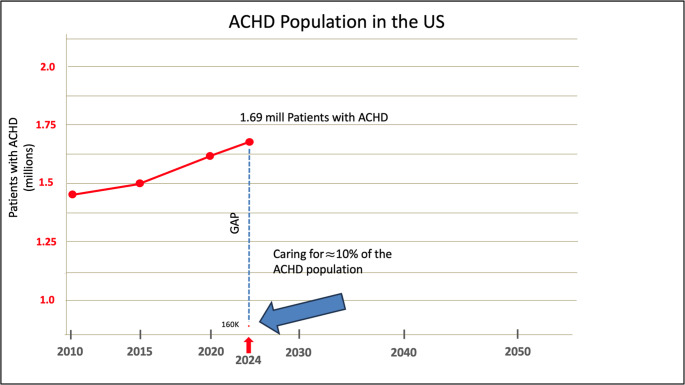
The care cap in the United States between the total number of adult congenital heart disease (ACHD) population and those receiving care within an Adult Congenital Heart Association Comprehensive Care Center

Currently available data only allows estimates of patients within ACHD CCC, and patients within these centers have the potential advantage of being cared for by multiple board-certified ACHD cardiologists, ACHD APP and nurses. It is expected that an ACHD practice with ACHD APP can manage a higher patient volume than a comparable practice without ACHD APP. ACHD APP may see follow up outpatient visits, communicate patient test results, perform inpatient consults, and therefore expand the program’s ability to care for more patients than an ACHD cardiologist alone. Thus, a care delivery model should consider that ACHD providers outside of an ACHA-accredited ACHD CCC may have less dedicated support and therefore could manage fewer than the ≤ 1000 patients per provider benchmark noted above. Based upon estimated data from ACHA, there are approximately 160,000 patients with ACHD receiving care at the 53 ACHD CCC. Given the current estimate of the ACHD population in 2024 at 1.69 million, ACHD CCC provide care for approximately only 10% of the ACHD population (Fig. 4).

In 2014, there were more than 1.4 million adults with CHD and only 63 ACHD cardiologists with formal or informal training in the US, yielding a patient to ACHD-trained provider ratio of 23,000:1. When board certification was offered in 2015, those 63 individuals that received informal and formal ACHD training were joined by several hundred cardiologists - mostly with pediatric cardiology backgrounds – who had no formal training but for whom ACHD had been a prominent part of their clinical practice. With the new ACHD board certification providing an additional tool for identification of ACHD workforce, the patient to provider ratio decreased. In 2024, with estimated 1.69 million patients with ACHD and 508 board certified ACHD cardiologists, the ratio of patients to board certified ACHD cardiologist was 3,300:1. If 508 board certified ACHD cardiologists were able to see approximately 950 patients per cardiologist, the workforce in 2024 would be able to care for 483,000 ACHD patients, representing 29% of the ACHD population, and highlighting a care gap for 71% of the ACHD population.

**Fig. 5:**
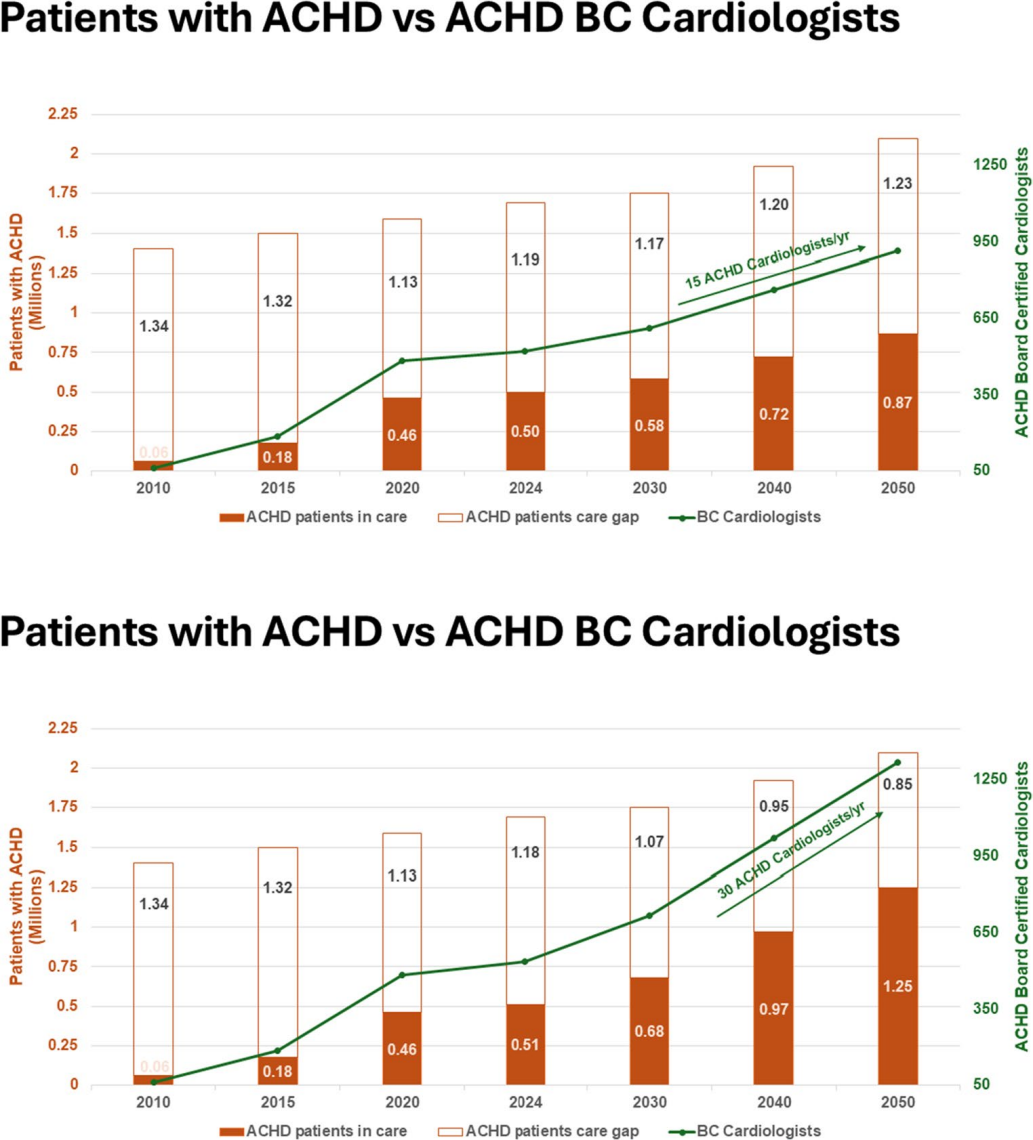
Adults with congenital heart disease (ACHD) vs ACHD Board Certified (BC) cardiologists. (A) Producing 15 ACHD cardiologists per year and (b) producing 30 ACHD cardiologists per year. With 15 cardiologists per year, the care gap improves but would cover less than 50% of the ACHD population in 2050. With 30 cardiologists year, 50% of the ACHD population would be under the care of a BC ACHD cardiologist by 2050

We then projected forward to the year 2050, using the benchmark of 950 ACHD patients per ACHD cardiologist. We generated two models: one in which an average of 15 ACHD cardiologists is produced per year, based on the average over the past 5 years; and a second in which we double the number of new ACHD cardiologists to 30 per year (Fig. 5). In both models, the gap between patients in ACHD specialized care and out of care persists over the next several decades. In the model with 30 new ACHD cardiologists per year, only 50% of the population would have access to an ACHD cardiologist in 2040 (Fig. 5 panel B). To reach the benchmark of ≤ 1000 patients per ACHD cardiologist by 2030 would require training 291 ACHD cardiologists per year; to reach this benchmark by 2040 would require training 120 ACHD cardiologists per year. Importantly, this projection does not account for ACHD cardiologists leaving the US workforce. A strategy focused solely on training more ACHD cardiologists thus will not resolve the care gap over the next few decades.

**Fig. 6:**
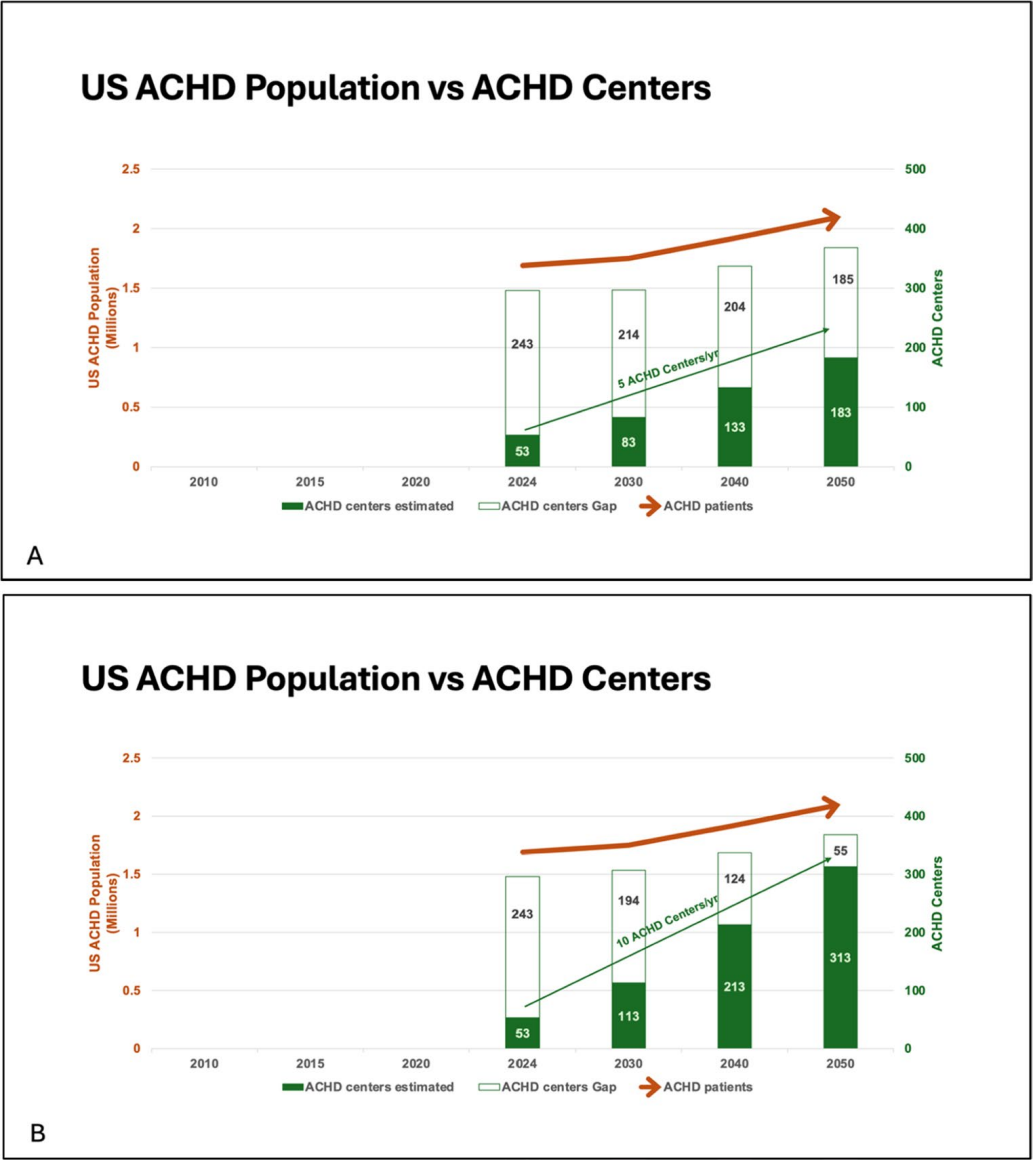
Adult congenital heart disease (ACHD) population in the United Statesand ACHD Centers. Projections of ACHD population growth vs number of ACHD Centers. If 5 new ACHD centers are created per year (**A**), approximately 50% of the ACHD population would be receiving care at an ACHD Center by 2050. If 10 new ACHD centers are created per year (**B**), the care gap would be nearly closed by 2050

Strategies to address the ACHD care gap must therefore include not only training more ACHD cardiologists, but also care delivery models and consideration of the population most in need of ACHD expertise. For example, if ACHD cardiologists focused on caring for those patients with (1) high CHD complexity, (2) moderate CHD complexity requiring yearly evaluations, and (3) newly diagnosed CHD, this would allow for about 50% of the ACHD population to be under the care of an ACHD cardiologist with the current workforce. Based on the concept previous described [18], an ACHD center caring for a regional general population of 1.9 million would care for approximately 2850 patients meeting one of these three criteria and could be staffed by 3 ACHD cardiologists. ACHD patients not meeting one of these criteria could potentially receive their care from a local ACHD clinic, or with a pediatric or adult cardiologist. Using this ACHD care model in which ACHD CCC cares only for the 50% of the ACHD patient population as defined above, the number of ACHD CCC would need to increase from 53 to 296 centers, and the number of ACHD cardiologists would need to increase from 508 to 888 to provide care for the US ACHD population in 2024. The care gap would be nearly closed by 2050 if there are 10 new ACHD centers per year and approximately 30 new ACHD cardiologists per year (Fig. 6). Again, this projection does not account for ACHD cardiologists inevitably leaving the workforce.

### Geography

**Fig. 7:**
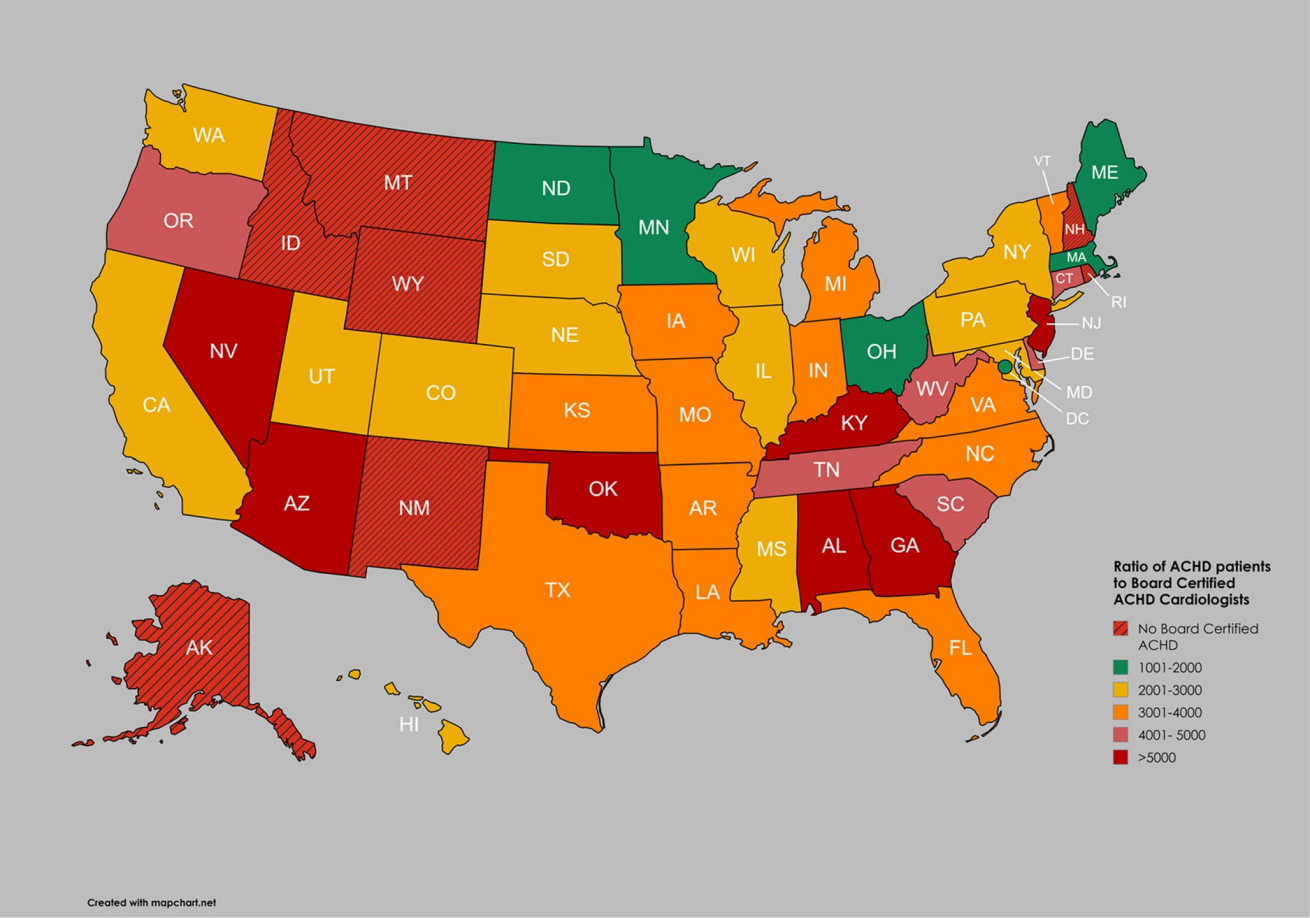
Ratio of adult congenital heart disease (ACHD) patients to board certified ACHD cardiologists by state. Five states have no ACHD cardiologists. There are no states that have board certified ACHD cardiologists and a ratio of ≤1000 ACHD patients per ACHD cardiologist, thus this is omitted from the figure legend. Five states and Washington, D.C. have an estimated 1001-2000 ACHD patients per ACHD cardiologist. Seven states have >5000 ACHD patients per ACHD cardiologist

Although there are 508 ACHD board certified cardiologists in the US, they are not evenly distributed in all states, and are not distributed in numbers consistent with population density per state. No state has an adequate number of cardiologists; some states have either no ACHD cardiologists, or the number of ACHD cardiologists is so small compared to the ACHD population that access to care is unattainable for much of the population (Fig. 7). State by state analysis is also important due to state insurance barriers to care.

**Fig. 8:**
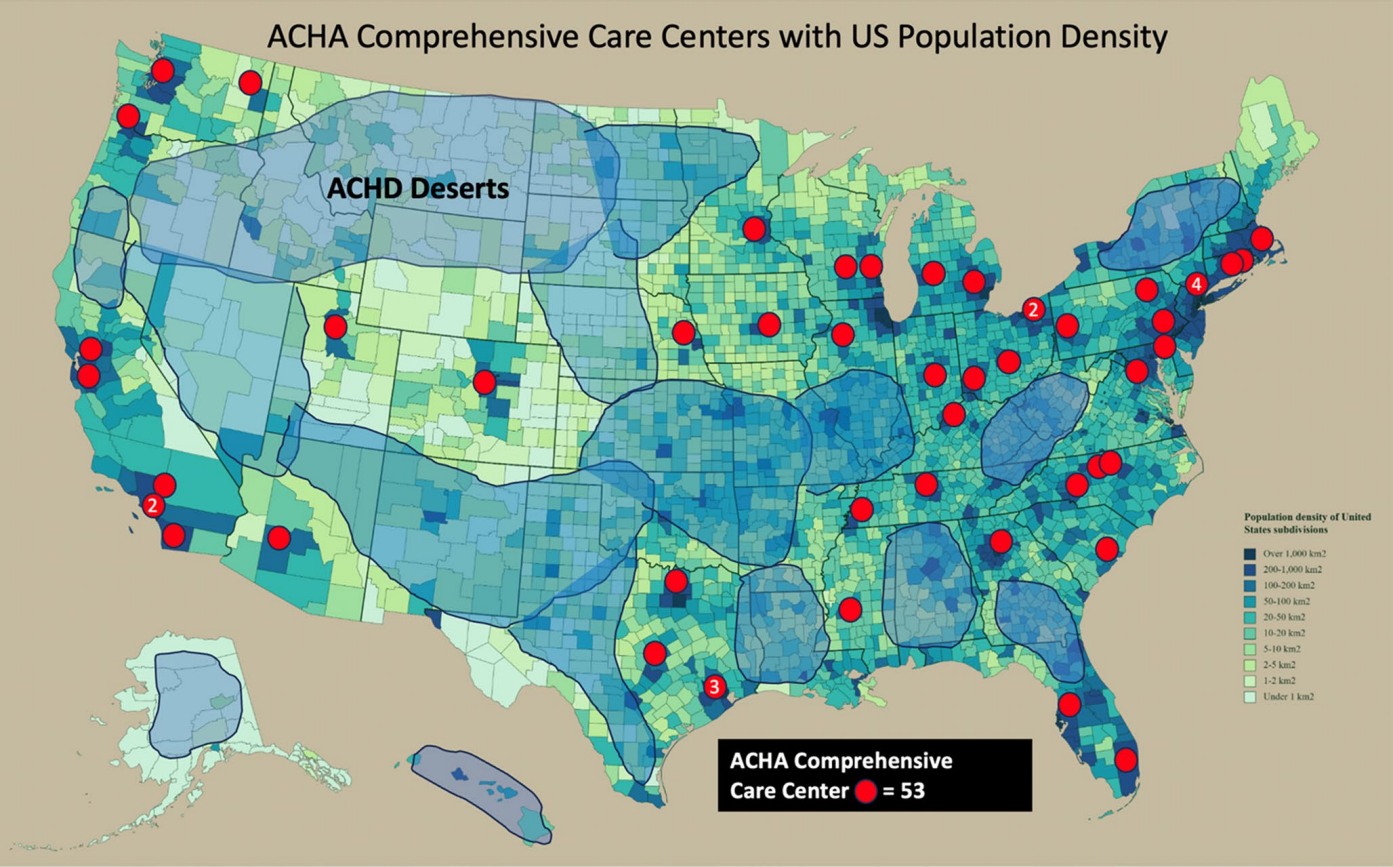
Adult Congenital Heart Association (ACHA) Comprehensive Care Centers with US population density. Large areas of the country lack geographic access to adults with congenital heart disease (ACHD) care centers

Beyond ACHD cardiologists and accredited centers, delivery of ACHD care is limited by US geography by state and by distance to an ACHD CCC or ACHD Clinic. There are large swaths of the US with tens of thousands of ACHD patients that live hundreds of miles from the closest ACHD CCC [9], as ACHD CCC have largely developed within large academic and urban areas (Fig. 8). Building additional ACHD centers in large cities provides access for those who live in close proximity but does not resolve the challenge of access for a large proportion of patients. Similarly, although training more ACHD cardiologists remains important, as 87% of the ACHD fellowship graduates in the past 5 years started their career at an existing ACHD CCC, new fellowship graduates are therefore not likely to significantly impact the current geographic barriers in access to care.

## The Future of ACHD Care

Given the considerations reviewed here, a multi-pronged solution is needed to meet the future patient care needs and improve access to ACHD care for a greater proportion of the population. To substantively address the ACHD care gap crisis will require the following action items:

1. Exposure to ACHD early in medical education. We conducted a national survey of internal medicine-pediatric (IM/Peds) residents (*n* = 52) to determine which factors influence career decisions. The study found that exposure to ACHD is either somewhat important (23%), a major factor (50%) or critically important (27%), with no resident stating it was not important. Unfortunately, most residents are not exposed to ACHD during their residency. Less than 10% of US IM residencies and less than 20% internal medicine cardiology programs provide any ACHD exposure.
  Most trainees entering either pediatric or adult cardiology fellowship have already developed an interest or focused pathway in general cardiology, imaging, interventional cardiology, electrophysiology, or heart failure. Therefore, stimulating trainee interest in ACHD needs to start early in medical training.
  We therefore encourage ACHD programs to:
  - Create opportunities for ACHD exposure for medical students through shadowing opportunities or interest groups. Similarly, for internal medicine, combined internal medicine/pediatrics, and pediatric residents, exposure to ACHD during didactic conferences, inpatient care or outpatient electives should be developed to provide exposure.
  - Offer away rotations in ACHD to trainees at institutions without ACHD programs.
  - Consider requiring rotations in ACHD for pediatric and adult cardiology fellows, preferably in their first or second year of fellowship, particularly at institutions with an ACHD fellowship or ACHD CCC.
2. Increase ACHD fellowship positions and continue to develop ACHD fellowship training programs. While there has been a steady increase in the number of ACHD fellowships over the past 10 years, further increases in the number of fellowship training positions may be needed to accommodate the increased demand for ACHD fellowship training as more trainees become interested in ACHD. Identifying hospital-based or external (i.e. philanthropic) sources of funding for ACHD fellowship positions will be essential. Also, as trainees are more likely to practice near their training location, developing new ACHD fellowships in areas without existing training program is important for addressing the care gap in less densely populated areas.
3. Address student debt. Student debt has increased consistently, with the average student debt exceeding $200,000 upon graduation from medical school according to the 2020 AAMC Physician Education Debt Report [19]. High student debt burden may discourage trainees from pursuing additional specialty training. Incentivizing ACHD fellowship training with loan repayment programs for trainees willing to practice ACHD in underserved areas may enable some trainees to pursue ACHD training.
4. Innovate medical education. The IM/Peds resident survey also found several additional factors influenced their career choice, with the top 3 being (a) length of training, (b) student debt, and (c) work/life balance. Length of training was the most important factor for 80% of the residents. When asked if they would prefer changing the traditional 3 years of adult cardiology or pediatric cardiology and 2 years of ACHD (3 + 2) to either (a) a model in which most of the adult or pediatric cardiology training is completed in 2 years, followed by 2 years of ACHD fellowship with a competency-based approach (“2 + 2” model), or (b) a model in which the adult or pediatric cardiology training remains 3 years but ACHD is shortened to 1 year (“3 + 1” model), 100% of the respondents preferred a 2 + 2 model.
  A proposal for a pilot program for accelerating training in adult cardiology and ACHD was submitted in 2024, approved by the ABIM and ACGME in 2025, and launched in July 2025. For trainees that are committed to a career in ACHD and demonstrate a favorable trajectory in adult cardiology milestones in their first year of adult cardiology fellowship, this accelerated training pathway compresses most of the adult cardiology fellowship clinical requirements into the first two years of fellowship, allowing them to effectively start ACHD fellowship training in year 3 at the same institution. This decreases the total length of training from 5 years to 4 years and allows for earlier entry into the workforce compared with the traditional pathway, thereby decreasing the financial burden of additional training for the trainee. The traditional 3 + 2 pathway remains an option for those who wish to pursue other experiences (i.e., advanced imaging) in year 3 of cardiology fellowship, those that undertake adult cardiology fellowship at an institution without an ACHD fellowship, those that decide in their 2nd year of cardiology fellowship to pursue ACHD training, or those that require the standard three years of adult cardiology fellowship to reach clinical competency in adult cardiology. Results from the adult cardiology-ACHD pilot program are anticipated in 2030. A similar proposal for American Board of Pediatrics and Pediatric Cardiology-ACHD is currently in review.
5. Develop additional ACHD Comprehensive Care Centers. High quality multi-disciplinary care within centers of excellence is necessary to improve outcomes and survival for patients with ACHD. These centers are also essential for trainee exposure, education, and research. However, we acknowledge that resources (congenital cardiac surgeons, interventionalists, and heart failure specialists, etc.) to build these centers may be a limiting factor.
6. Create additional ACHD care networks, and expand exisiting ACHD CCCnetworks. In this hub-and-spoke model, ACHD CCC serves as the central “hub,” and the “spokes” can be any of the following:
  - Satellite clinics operated by the same institution as the ACHD CCC. Many large academic centers now have satellite locations that can include ACHD clinics.
  - ACHD Clinics operated by a different institution from the ACHD CCC
  - Adult cardiology or pediatric cardiology clinics not listed in the ACHA ACHD Clinic Directory, in which ACHD patients are seen. These may be operated by the same institution or a different institution as the ACHD CCC.
    These“spokes” are critical for access to care for patients that are distant from an ACHD CCC, as many have socioeconomic barriers that prevent them from traveling to the ACHD CCC. Partnerships between ACHD CCC and their “spokes” allow expansion of high quality ACHD care into moderate size communities and geographic ACHD deserts and provide access to ACHD CCC resources such as complex imaging, catheter-based-interventions, and CT surgery.
    Once ACHD patients are seen at one of these clinics, patients are often referred to the ACHD CCC for ACHD initial evaluation and care. However, some patients with simple or moderate complexity ACHD may be followed longitudinally at the satellite clinic with only periodic check-ins at the ACHD CCC. Collaborative Care Models, in which ACHD cardiologists in ACHD CCC or ACHD Clinics partner with local general cardiologists and pediatric cardiologists, can also improve access to care for geographically isolated patients. A Collaborative Care Model has been implemented successfully in Hawaii to provide care to ACHD patients across the state, providing proof of feasibility for other areas with similar challenges with access to care.
    Partnerships between ACHD CCC and ACHD Clinics alone would yield 355 points of access for patients with ACHD in the US, and has the potential to maintain high quality ACHD care. ACHA and stakeholder organizations should support development of ACHD care networks with quality-based criteria for ACHD CCC and ACHD Clinics.
7. Innovate the ACHD care models to include trained ACHD APP. To reach a greater proportion of the population, we will need to embrace the fact that the ACHD care gap is too great to be met by only focusing on producing more ACHD cardiologists.
8. Improve access to telehealth. During the COVID-19 pandemic, there was a widespread uptake of telehealth across the US. This rapid adoption of telehealth by hospital systems, payors, providers, and patients can enable ACHD care in geographically remote areas and decrease the travel burden for patients and providers. Maintaining access to telehealth in the post-pandemic era and ensuring appropriate reimbursement for ACHD telehealth consistent with time, effort and subspecialty care are critical.
9. Improve reimbursement for ACHD specialty care. The discrepancy in reimbursement and salary support for ACHD cardiologists needs to be addressed to incentivize selecting ACHD for residents and fellows who are interested in ACHD but are socioeconomically challenged by the length of training compared to salary support. It is also important to address for provider retention in the field. This may be achieved by reviewing relative value units (RVUs) attached to the complexities of ACHD activities, and by designating ACHD as a separate specialty by Association of American Medical Colleges (AAMC).

## Conclusions

As the ACHD population grows, it is critical that we consider a multi-pronged approach to meet the increased need for specialty care. This includes measures to increase the ACHD workforce by providing opportunities for medical students and residents to learn about ACHD, increasing ACHD fellowship opportunities and decreasing the financial burden of additional training. Other measures are expanding APP roles in ACHD, and consideration of innovative approach to care delivery models that includes collaboration between ACHD CCC and ACHD clinics, general cardiologists, and pediatric cardiologists.

## Data Availability

Data would be provided under request through the publisher and approved by the publisher in consultation with the senior author Daniels.

## References

[1] Marelli AJ, Ionescu-Ittu R, Mackie AS, et al. Lifetime prevalence of congenital heart disease in the general population from 2000 to 2010. Circulation. 2014;130(9):749–56. 24944314 10.1161/CIRCULATIONAHA.113.008396

[2] Benziger CP, Stout K, Zaragoza-Macias E et al. Projected growth of the adult congenital heart disease population in the united States to 2050: an integrative systems modeling approach. Popul Health Metr 2015;13–29. 10.1186/s12963-015-0063-z PMC4606959 26472940

[3] Baumgartner H. Geriatric congenital heart disease: a new challenge in the care of adults with congenital heart disease. Eur Heart J. 2014;35:683–5. 24014388 10.1093/eurheartj/eht358

[4] Koyak Z, Harris L, de Groot JR, Silversides CK, Oechslin EN, Bouma BJ, Budts W, Zwinderman AH, Van Gelder IC, Mulder BJ. Sudden cardiac death in adult congenital heart disease. Circulation. 2012;126(16):1944–54. 22991410 10.1161/CIRCULATIONAHA.112.104786

[5] Verheugt CL, Uiterwaal CS, van der Velde ET, et al. The emerging burden of hospital admissions of adults with congenital heart disease. Heart. 2010;96(11):872–8. 20406765 10.1136/hrt.2009.185595

[6] Verheugt CL, Uiterwaal CS, van der Velde ET, et al. Mortality in adult congenital heart disease. Eur Heart J. 2010;31(10):1220–9. 20207625 10.1093/eurheartj/ehq032

[7] Maxwell BG, Wong JK, Lobato RL. Perioperative morbidity and mortality after noncardiac surgery in young adults with congenital or early acquired heart disease: a retrospective cohort analysis of the National surgical quality improvement program database. Am Surg. 2014;80(4):321–6. 24887660

[8] Agasthi P, Van Houten HK, Yao X, Jain CC, Egbe A, Warnes CA, et al. Mortality and morbidity of heart failure hospitalization in adult patients with congenital heart disease. J Am Heart Assoc. 2023. 10.1161/JAHA.123.030649. 38018491 10.1161/JAHA.123.030649 PMC10727341

[9] Mylotte D, Pilote L, Ionescu-Ittu R, et al. Specialized adult congenital heart disease care: the impact of policy on mortality. Circulation. 2014;129(18):1804–12. 24589851 10.1161/CIRCULATIONAHA.113.005817

[10] Nguyen VP, Dolgner SJ, Dardas TF, et al. Improved outcome of heart transplantation in adults with congenital heart disease receiving regionalized care. J Am Coll Cardiol. 2019;74(23):2908–18. 31806135 10.1016/j.jacc.2019.09.062

[11] Plappert L, Edwards S, Senatore A, De Martini A. The epidemiology of persons living with Fontan in 2020 and projections for 2030: development of an epidemiology model providing multinational estimates. Adv Ther. 2022;39(2):1004–15. 34936056 10.1007/s12325-021-02002-3 PMC8866255

[12] Gurvitz M, Dunn JE, Bhatt A, Book WM, Glidewell J, Hogue C, et al. Characteristics of adults with congenital heart defects in the united States. J Am Coll Cardiol. 2020;76(2):175–82. 32646567 10.1016/j.jacc.2020.05.025 PMC9082530

[13] Gilboa SM, Devine OJ, Kucik JE, Oster ME, Riehle-Colarusso T, Nembhard WN, et al. Congenital heart defects in the United States: estimating the magnitude of the affected population in 2010. Circulation. 2016;134(2):101–9. 27382105 10.1161/CIRCULATIONAHA.115.019307 PMC4942347

[14] Landzberg MJ, Murphy D, Davidson WR, et al. Task force 4: organization of delivery systems for adult congenital heart disease. J Am Coll Cardiol. 2001;37(5):1161–98. 10.1016/s0735-1097(01)01275-x 11300421

[15] https://www.acgme.org/globalassets/PFAssets/Presentations/153_adult_congenital_heart_disease_07012015_1-YR.pdf

[16] https://www.achaheart.org/media/3471/accreditationprogramcriteria2022.pdf

[17] https://www.achaheart.org/your-heart/resources/clinic-directory/

[18] Marelli AJ, Therrien J, Mackie AS, et al. Planning the specialized care of adult congenital heart disease patients: from numbers to guidelines; an epidemiologic approach. Am Heart J. 2009;157(1):1–8. 19081390 10.1016/j.ahj.2008.08.029

[19] Youngclaus J, Fresne JA. Physician education debt and the cost to attend medical school: 2020 update. Washington (DC): Association of American Medical Colleges; 2020. Available from:https://www.aamc.org/data-reports/students-residents/report/physician-education-debt-and-cost-attend-medical-school

